# *Notes from the Field:* Severe *Bartonella quintana* Infections Among Persons Experiencing Unsheltered Homelessness — New York City, January 2020–December 2022

**DOI:** 10.15585/mmwr.mm7242a3

**Published:** 2023-10-20

**Authors:** Shannan N. Rich, Amy Beeson, Leah Seifu, Kara Mitchell, Danielle Wroblewski, Stefan Juretschko, Marina Keller, Rachel Gnanaprakasam, Mariam Agladze, Rich Kodama, Tania Kupferman, Julu Bhatnagar, Roosecelis B. Martines, Sarah Reagan-Steiner, Sally Slavinski, Matthew J. Kuehnert, Camille Bergeron-Parent, Gabriella Corvese, Grace E. Marx, Joel Ackelsberg

**Affiliations:** ^1^Epidemic Intelligence Service, CDC; ^2^Division of Vector-Borne Diseases, National Center for Emerging and Zoonotic Infectious Diseases, CDC; ^3^New York City Department of Health and Mental Hygiene, New York, New York; ^4^New York State Department of Health Wadsworth Center; ^5^Northwell Health Laboratories, Little Neck, New York; ^6^Westchester Medical Center, Valhalla, New York; ^7^Bellevue Hospital Center, New York, New York; ^8^Memorial Sloan Kettering Cancer Center, New York, New York; ^9^Division of High-Consequence Pathogens and Pathology, National Center for Emerging and Zoonotic Infectious Diseases, CDC; ^10^Office of the Director, National Center for Emerging and Zoonotic Diseases, CDC; ^11^New York City Department of Homeless Services, New York, New York.

*Bartonella quintana* infection is a vectorborne disease transmitted by the human body louse (*1*). In the United States, homelessness is the principal risk factor for *B. quintana* infection (*2*), likely attributable to limited access to hygiene facilities (*1*). This infection is not nationally notifiable in the United States, and its incidence is unknown. Acute *B. quintana* infection can cause fever, headache, and bone pain; severe manifestations include chronic bacteremia, bacillary angiomatosis, and infective endocarditis (*3*). Because the bacterium requires special conditions to grow in culture, standard blood cultures are usually negative (*4*). Diagnosis by serology is most common; however, cross-reactivity with other *Bartonella* species (e.g., *B. henselae*) can hamper interpretation. Molecular assays specific for *B. quintana* have been developed (*5*), but availability is limited to a few laboratories. Once diagnosed, infection can be cured by several weeks to months of antibiotic therapy.

## Investigation and Outcomes

In January and April 2023, the New York City (NYC) Department of Health and Mental Hygiene (DOHMH) was alerted to two cases of *B. quintana* infection that occurred during 2022 among persons who had experienced unsheltered homelessness in NYC and later died (one died because of the infection, and the other because of an unrelated cause). DOHMH conducted retrospective active surveillance within clinical laboratories of five large NYC hospital networks to identify additional cases with culture, molecular, or serologic laboratory results for *B. quintana* or *Bartonella* spp. and reviewed electronic medical records of all identified patients. One patient’s family provided clinical outcome details not found in the medical record. Housing status was determined from the medical record or the NYC Department of Homeless Services (DHS) system. This activity was reviewed by CDC, deemed not research, and was conducted consistent with applicable federal law and CDC policy.^†^

Four additional cases were identified that occurred in NYC during January 2020–December 2022 (Table). Four of the six total cases occurred during 2022. Five patients received a positive molecular diagnostic test result specific for *B. quintana*^§^; one received a positive *Bartonella* polymerase chain reaction (PCR) test result not specific to *B. quintana*. Five patients were hospitalized for complications of *B. quintana* infection; the median duration of hospitalization was 34 days (range = 8–78 days). Four patients received a diagnosis of culture-negative, left-sided endocarditis and underwent surgical valve replacement; three experienced renal failure, and two died from endocarditis-related complications. One patient died from complications of traumatic injury not related to *B. quintana* infection. All six patients had experienced recent unsheltered homelessness either at the time of hospitalization (four) or within the preceding year (two) and had incidental contact or no contact with the NYC DHS shelter or outreach system; five had a documented mental health or substance use disorder; no cases were epidemiologically linked.

**TABLE:** Demographic information, clinical features, risk factors, and outcomes of six persons experiencing homelessness who received a diagnosis of *Bartonella quintana* infection — New York City, January 2020–December 2022.

Characteristic	No. (%)
**Age, yrs, mean (range)**	52 (38–69)
**Sex**	
Female	1 (17)
Male	5 (83)
Diagnosis*	
Aortic valve endocarditis	4 (67)
Mitral valve endocarditis	1 (17)
Bacillary angiomatosis	1 (17)
Bacteremia	1 (17)
**History of unsheltered homelessness**	6 (100)
**History of alcohol use**	5 (83)
**History of mental health condition**	3 (50)
**HIV infection** ^†^	1 (17)
**Hospital admission, yr**	
2020	1 (17)
2021	1 (17)
2022	4 (67)
**Outcome**	
Traumatic injury–related death	1 (17)
Endocarditis-related death	2 (33)
Renal failure	3 (50)

* One patient received two diagnoses. ^†^ CD4 T-cell count <50 cells/mm^3^ at time of diagnosis.

## Preliminary Conclusions and Actions

*B. quintana* infection can result in severe outcomes, including death, and incur substantial health care costs from prolonged hospitalizations and surgical interventions. The total number of *B. quintana* cases is likely higher than what is reported here for several reasons: 1) persons experiencing unsheltered homelessness often do not seek health care services, 2) health care providers are less likely to consider bartonellosis in patients without severe disease, and 3) laboratory diagnosis is challenging.

To help identify patients at risk for *B. quintana* infection, clinicians should consider housing status. Persons experiencing homelessness who have mental health conditions or substance use disorders might be less likely to access preventive hygiene services. Clinicians and health care systems could increase diagnostic and treatment support for behavioral health conditions in this population to prevent serious medical conditions, including bartonellosis resulting from body louse infestation. In addition, it is important that patients with a history of unsheltered homelessness and either prolonged subjective fevers without a known etiology, a vasoproliferative skin rash, or a diagnosis of culture-negative endocarditis be tested for *B. quintana* infection with a molecular diagnostic laboratory assay and considered for empiric treatment in consultation with an infectious disease physician.

## Footnotes

† 45 C.F.R. part 46, 21 C.F.R. part 56; 42 U.S.C. Sect. 241(d); 5 U.S.C. Sect. 552a; 44 U.S.C. Sect. 3501 et seq.

§ Three patients received a diagnosis of *B. quintana* based on reverse transcriptase–PCR testing and molecular sequencing at the New York State Department of Health Wadsworth Center; two patients received a diagnosis based on *B. quintana* PCR testing and molecular sequencing of retrospectively identified fixed tissue samples at CDC’s Infectious Disease Pathology Branch.
